# Research Progress of Pancreas-Related Microorganisms and Pancreatic Cancer

**DOI:** 10.3389/fonc.2020.604531

**Published:** 2021-01-14

**Authors:** Wenqing Zhang, Kunpeng Zhang, Peng Zhang, Juanjuan Zheng, Congcong Min, Xiaoyu Li

**Affiliations:** Department of Gastroenterology, The Affiliated Hospital of Qingdao University, Qingdao, China

**Keywords:** microbial diversity, pancreatic cancer, inflammatory, immunity, metabolism

## Abstract

Pancreatic cancer is one of the most common digestive system cancers. Early diagnosis is difficult owing to the lack of specific symptoms and reliable biomarkers. The cause of pancreatic cancer remains ambiguous. Smoking, drinking, new-onset diabetes, and chronic pancreatitis have been proven to be associated with the occurrence of pancreatic cancer. In recent years, a large number of studies have clarified that a variety of microorganisms colonized in pancreatic cancer tissues are also closely related to the occurrence and development of pancreatic cancer, and the specific mechanisms include inflammatory induction, immune regulation, metabolism, and microenvironment changes caused by microorganism. The mechanism of action of the pancreatic colonized microbiome in the tumor microenvironment, as well as immunotherapy approaches require further study in order to find more evidence to explain the complex relationship between the pancreatic colonized microbiome and PDAC. Relevant studies targeting the microbiome may provide insight into the mechanisms of PDAC development and progression, improving treatment effectiveness and overall patient prognosis. In this article, we focus on the research relating to the microorganisms colonized in pancreatic cancer tissues, including viruses, bacteria, and fungi. We also highlight the microbial diversity in the occurrence, invasion, metastasis, treatment, and prognosis of pancreatic cancer in order to elucidate its significance in the early diagnosis and new therapeutic treatment of pancreatic cancer, which urgently need to be improved in clinical practice. The elimination or increase in diversity of the pancreatic microbiome is beneficial for prolonging the survival of PDAC patients, improving the response to chemotherapy drugs, and reducing tumor burden. The colonization of microorganisms in the pancreas may become a new hotspot in the diagnosis and treatment of pancreatic cancer.

## Introduction

Pancreatic cancer is the most refractory malignant tumor, owing to its lack of early diagnostic markers, early tendency for neurovascular invasion, rapid deterioration, extensive metastasis to multiple organs, high postoperative recurrence rate, high postoperative distant metastasis rate, poor response to chemoradiotherapy, and several additional characteristics, which lead to an extremely poor prognosis (1). Following decades of improvements in surgical techniques, radiotherapy, chemotherapy, and biotherapy, the five-year survival rate has barely increased, and now stands at 9% (2). Known genetic drivers in the etiology of pancreatic cancer include KRAS, TP53, BRCA, etc. Family history (3), chronic pancreatitis (4, 5), obesity (6), diabetes (7, 8), smoking (9), and heavy drinking (10) are all risk factors for pancreatic cancer. Although ultrasound endoscopy and ultrasound-guided fine-needle aspiration have brought hope for the early diagnosis of pancreatic cancer in recent years (11), further progress is needed to identify specific biomarkers and improve the early diagnosis rate.

The human microbiome is a new target for studying cancer development and treatment. It can be directly carcinogenic by promoting an inflammatory response, and may also play an anti-cancer role by changing the tumor immune microenvironment (12). Interactions between microorganisms near or far from tumor tissues affect cancer progression and disease progression in specific ways (12). Studies have shown that each tumor type has a distinct microbiome composition, and that most of the bacteria in the tumor are intracellular and exist in cancer cells and immune cells (13). The same is true for pancreatic cancer. New research suggests bacteria may be playing a role in other gastrointestinal cancers (14). However, the latest research indicates that the specific bacterial ecosystem in the pancreatic cystfluid sample may reflect the local microbiota in the pancreas (15). In contrast to normal pancreatic tissue, a large number of microorganisms, such as bacteria and fungi, colonize the pancreatic cancer tissue. Their presence not only promotes the occurrence and development of pancreatic cancer, but also affects the response and prognosis of pancreatic cancer to treatment (16, 17). **Table 1** has summarized some studies on pancreatic colonizing microorganisms and pancreatic cancer. This article aims to analyze the relationship between the colonization of microbes in pancreatic cancer tissue and the occurrence and development of pancreatic cancer.

**Table 1:** Research on microorganism and pancreatic cancer in pancreas and pancreatic cancer.

Time	Author	Methods	Conclusions	Magazine	Reference
2019	Del Castillo, E et al.	16S rRNA gene sequencing	Bacterial DNA profiles in the pancreas were similar to those in the duodenum tissue of the same subjects	Cancer Epidemiol Biomarkers Prev	(20)
2018	Pushalkar, S et al.	16S rRNA gene sequencing	Cancerous pancreas has a significantly richer microbiome	Cancer Discov	(16)
2019	Riquelme, E et al.	16S rRNA gene sequencing	Higher alpha diversity found in the tumor microbiome of LTS patients is associated with long-term survival	Cell	(17)
2017	Geller, LT et al.	16S rRNA gene sequencing	Of the 113 human PDACs that were tested, 86 (76%) were positive for bacteria, mainly Gammaproteobacteria.	Science	(21)
2020	Gnanasekaran J et al.	16S rRNA gene sequencing	*P. gingivalis* survives inside pancreatic cancer cells.	Cancers (Basel)	(24)
2015	Mitsuhashi, K et al.	custom-made TaqMan primer/probe sets	Fusobacterium species were detected in pancreatic cancer tissue.	Oncotarget.	(26)
2018	Maekawa T et al.	16S rRNA gene sequencing	*Enterococcus faecalis* was detected in pancreatic tissue from chronic pancreatitis and pancreatic cancer patients.	Biochem Biophys Res Commun	(38)
2019	Aykut, B et al.	The modified Illumina metagenomics protocol	pancreatic ductal adenocarcinoma (PDA) tumours in humans and mouse models of this cancer displayed an increase in fungi of about 3,000-fold compared to normal pancreatic tissue.	Nature	(51)
2013	Jin Y, et al.	Detection of HBV covalently closed circular DNA	HBsAg and HBcAg were expressed in 21.0% (34/162) of PC and 29.0% (47/162) of non-tumor pancreatic tissues.	Cancer Lett	(67)

## Composition of Microorganisms Colonized in Pancreatic Cancer Tissue

### Bacterial Colonization in Pancreatic Cancer Tissue

**Figure 1:**
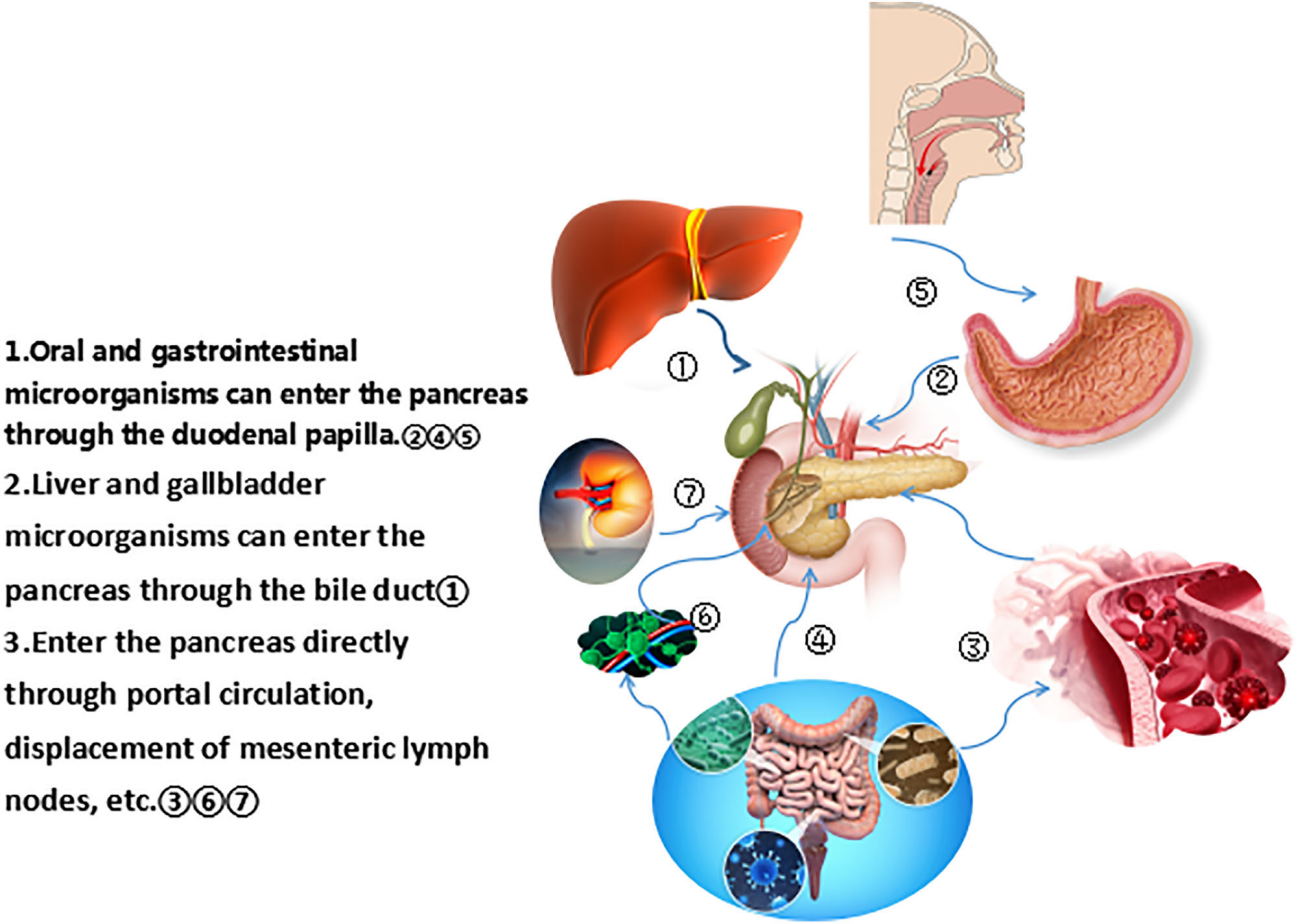
Different ways for microorganisms from different parts to enter the pancreas.

The common hepatic duct and the pancreatic duct merge to form the common bile duct, which opens to the major duodenal nipple. The migration of bacteria from the oral cavity and gastrointestinal tract to the pancreas through the pancreatic duct is the source of bacteria in the pancreatic tissue. In cats, E coli can spread to the pancreas by the blood stream, transmurally from the colon, and enter the pancreatic duct through reflux. Pathogens may spread from the colon, gallbladder, and kidneys to the pancreas (18). The way bacteria enter the pancreas is still controversial, and may include some mechanisms, such as oral route, via translocation from the lower gastrointestinal tract through the portal circulation or mesenteric lymph nodes (19). **Figure 1** lists the different ways that microorganisms from different parts of the pancreas enter the pancreas. Regardless of the disease state, the bacterial DNA profile in the pancreas is similar to that in the duodenum of the same subject, suggesting that bacteria may migrate from the intestine to the pancreas. Pancreatic colonized microbiota, as well as gut microbiota, are related to disease development (19, 20). Riquelme et al. (17) compared the microbiome with similar specimens and found that the human gut microbiome accounted for 25% of the human tumor microbiome, while the bacterial composition in normal adjacent tissues was different from that in tumors, which indicated the ability of the gut microbiota to specifically colonize pancreatic tumors. Studies have found that the bacterial composition in pancreatic cancer tissue is different from that in normal pancreatic tissue. Pushalkar et al. (16) sequenced the multivariate region of the bacterial 16S ribosomal RNA (rRNA) gene in 12 pancreatic ductal adenocarcinoma (PDAC) tissues and detected 13 different phyla, of which *Proteu*s (45%), *Bacteroides* (31%), and *Firmicutes* (22%) were relatively high and present in all specimens. Actinobacteria (1%), although low in content, were also prevalent in all specimens. In addition, the genera Pseudomonas and *Elizabethkingia* were also highly abundant. Geller et al. (21) performed rRNA fluorescence in situ hybridization with probes targeting bacterial 16S rRNA and deep sequencing of polymerase chain reaction (PCR)-amplified bacterial 16S rDNA of 65 PDAC tumor tissues. The most common group identified in pancreatic cancer tissues was the class *Gammaproteobacteria*, most of which were Enterobacteriaceae and Pseudomonas, which demonstrated that bacteria colonized the pancreas and were components of the pancreatic cancer tumor microenvironment.

Oral microbial community composition is associated with pancreatic cancer (22, 23). Gnanasekaran, J., et al found that *Porphyromonas gingivalis* survives inside pancreatic cancer cells. This property can be enhanced in vitro and increased by hypoxia, which is the main feature of pancreatic cancer. Increased tumor cell proliferation was related to the degree of intracellular persistence, and infection of tumor cells with *P. gingivalis* led to enhanced growth *in vivo* (24). Oral microorganisms may colonize the pancreas through the gastrointestinal tract, especially in the case of pancreatitis, the microbiota isolated from the pancreas is similar to the oral microbiota (25). A certain number of Clostridium species that originally colonized the oral cavity can be detected in normal pancreatic tissues (26). Compared with non-cancer patients, patients with ampullary cancer or pancreatic ductal adenocarcinoma have significantly reduced numbers of *Lactobacillus*, while oral bacteria such as *Porphyromonas*, *Clostridium*, and *Prevotella* are more abundant (20).

The relationship between *Helicobacter pylori* and pancreatic cancer remains controversial. To date, numerous case-control studies, prospective cohort studies, and meta-analyses have suggested that *H. pylori* infection is associated with an increased risk of PDAC (27–29). However, some studies have found that there is no correlation between the two (30–32). A Swedish study detected *H. pylori* DNA in pancreatic tumor tissues and/or surrounding tissues in 60% of patients with pancreatic cancer, proposing that *H. pylori* may play a role in the occurrence of pancreatic cancer (33). It has also been found that *H. pylori* DNA cannot be detected in pancreatic juice or pancreatic tissues in chronic pancreatitis and PDAC (34), suggesting that *H. pylori* does not colonize the pancreas directly and may trigger pancreatic carcinogenesis in an indirect manner. The possible mechanisms are as follows: the colonization of *H. pylori* in the gastric antrum increases gastric acid secretion, decreases somatostatin production, increases pancreatic bicarbonate secretion, promotes pancreatic hyperplasia, and accelerates DNA synthesis. *H. pylori* colonize the gastric body, and bacterial overgrowth causes an increase in the production of bacteria-catalyzed N-nitrosamines, which act on the pancreas through the blood. Long-term pancreatic cell proliferation and the stimulation of endogenous carcinogen N-nitroso compounds, together with the reduction of DNA repair ability, lead to the occurrence and development of pancreatic cancer (35–37). Whether *H. pylori* colonize the pancreas and the impact of colonization on the immune microenvironment of pancreatic tumors warrants further investigation.

Bile is associated with bacterial colonization of the pancreas. A study on chronic pancreatitis and pancreatic cancer found that 29 pancreatic juice samples collected from 20 patients with pancreatic cancer and 16 patients with duodenal cancer or bile duct cancer, using drainage tubes following pancreatectomy, tested positive for enterococcal DNA (38). *Enterococcus* and *Enterobacter* species were also detected in the bile, and *Enterococcus faecalis* was detected in the pancreatic tissue of patients with chronic pancreatitis and pancreatic cancer (38). Bile duct obstruction and liver damage also affect the microbiome. PC-related liver damage disrupts the normal gut balance, driving reductions in multiple normal gut-residing bacteria (39). It is suggested that bile microbiota such as *Escherichia coli* may affect the pancreatic microbiota. Some specific bacteria can migrate from the gallbladder to the pancreas, and the clearance of these bacteria may trigger the Th1 type immune response, which can play a protective effect on the growth of pancreatic cancer (40–42). Bacterial DNA was detected in the duodenal fluid collected from patients with biliary obstruction after common bile duct stent placement by endoscopy (n = 6), otherwise there was almost no bacterial DNA was found in the control group without stent placement (n = 5). It is suggested that the common bile duct stent may affect the duodenal microbiome. Many clinical factors such as proton pump inhibitors may affect the composition of colonized microbiome in the pancreas and increase the risk of pancreatic cancer (43, 44).

### Fungal Colonization in Pancreatic Cancer Tissue

Owing to the low abundance of intestinal fungi and the lack of a well-characterized reference genome, the action of fungal flora in pancreatic cancer progression is a relatively new and undiscovered field. The migration of certain fungi from the intestine to colonize the pancreas is related to the occurrence of pancreatic cancer. The fungal genome may be a new target for the treatment of pancreatic cancer (45, 46). A study in Taiwan showed that *Candida* infection (CI) can significantly increase overall and certain individual cancer risks (47). *Candida* can produce compounds such as nitrosamines, which are identified carcinogens that play a role in oral cancer initiation (48, 49) A previous study suggested that *Candida albicans* promotes cancer through a proinflammatory response, mediated by an increase in cytokine production and adhesion-molecule expression (50).

Aykut et al. introduced fungal strains labeled with green fluorescent protein (GFP) into the intestine of mice. Fungi migrated to the pancreas within 30 min, indicating that the intestinal fungi may colonize the pancreas (51). The fungi in the pancreatic tumors of human and mouse models were 3,000 times more than normal pancreatic tissues and contained several different species. For example, the most common species of the pancreas in KC mice was Malassezia, which had a significant increase in relative abundance compared to the intestine (51). Pathogenic fungi in pancreatic tumor tissues bind Mannose-binding lectin (MBL) to activate the complement C3 cascade and promote pancreatic cancer progression. MBL is a soluble lectin of the innate immune system that is produced by the liver and secreted into the circulatory system to activate the lectin complement pathway, enhance the phagocytosis of microorganisms by leukocytes, and regulate inflammation (52). The tumor microenvironment (TME) plays a key role in tumorigenesis, development, metastasis, and recurrence. Complement activation in TME has immunomodulatory functions, and the interactions between the complement system and cancer cells contribute to proliferation, epithelial–mesenchymal transition, migration, and invasion of tumor cells (53, 54). MBL recognizes the carbohydrate structure produced by Malassezia and activates protein C3, triggering the complement cascade inflammatory immune response. Complement activation stimulates cell proliferation and migration and promotes tumor growth (55). Deletion of MBL/C3 in the extratumoral compartment or knockdown of C3aR in tumor cells can inhibit tumor growth. Therefore, further studies on the role of fungal flora as a potential prognostic tool for early diagnosis of this cancer are warranted.

### Viral Colonization in Pancreatic Cancer Tissue

Some case-control studies and meta-analyses have shown that hepatitis B or C virus infection increases the risk of pancreatic cancer (56–59). However, some studies have shown that hepatitis B and C virus infections are not a risk factor for pancreatic cancer (60–62). A Swedish study showed that HCV infection may be associated with an increased risk of pancreatic cancer (63). There are also data in China showing that chronic hepatitis B and hepatitis B carriers (HBsAg-positive) have a significantly increased risk of pancreatic cancer (64). Hoefs et al. reported for the first time in 1980 that HBsAg was detected in the pancreatic juice of HBV infected patients (65), HBsAg and HBcAg were detected in the cytoplasm of pancreatic acinar cells (66). Studies have confirmed that HBV not only infects pancreatic tissues of pancreatic cancer patients but also replicates in pancreatic tissues. Chronic inflammation caused by HBV infection may play a role in the development of pancreatic cancer (67). Hepatitis B virus encodes the regulatory HBx protein that promotes transcription of the viral genome (68) and causes prolonged metabolic disorders, all of which may promote the development of pancreatic cancer (67). The integration of HBV/HCV DNA with the DNA of infected cells delays the clearance of the host immune system of HBV/HCV (69). Although the HBV replication level of pancreatic cancer cells are very low (67), the potential role of hepatitis virus in chronic pancreatitis and pancreatic cancer cannot be ignored.

## Pancreatic Related Microbe-Induced Inflammation and Pancreatic Cancer

Inflammation triggered by microorganisms is beneficial in defending against pathogens; however, if inflammation persists, it may lead to tissue fibrosis and even carcinogenesis, Microbial-induced inflammation leads to tumor development by activating tumor-related inflammatory signaling pathways, including proinflammatory cytokines, Toll-like receptor (TLR)/MyD88 (myeloid differentiation primary response gene 88) pathway, nuclear factor-kappa B (NF-κB), etc., constitute a fine and complex network (70–72). Toll-like receptors (TLRs) are usually expressed on immune cells such as macrophages, dendritic cells, mast cells, as well as on eosinophils and some epithelial cells. They play a central role in the recognition of harmful molecules that belong to invading microorganisms or internal damaged tissues, which lead to inflammation. Microorganisms colonized in the pancreas may cause inflammation in this way, thereby promoting tumorigenesis (73). Lipopolysaccharide (LPS) is a component of the cell wall of Gram-negative bacteria and can be specifically recognized by Toll-like receptor 4 (TLR4), a family member of the pattern recognition receptor (PRR) (74). Atsuo Ochi et al. show that lipopolysaccharide can promote pancreatic tumorigenesis, whereas TLR4 inhibition is protective. In addition, blockade of the MyD88-independent TRIF pathway is protective against pancreatic cancer, whereas blockade of the MyD88-dependent pathway surprisingly exacerbates pancreatic inflammation and malignant progression (74). Pattern recognition receptors (PRRs), such as Toll-like receptors (TLRs) located on monocytes or endolysosome membranes, interact with pathogen-related or risk-related molecular patterns to trigger mitogen-related protein kinases (MAPK) kinase-kinase TAK1 (transforming growth factor β activated kinase 1) and IκB (inhibitor of nuclear factor κB) kinase IKK signaling pathway (70). The main reaction between inflammation and cancer is the imbalance of oxidative stress (75). Pathogenic microbes often activate the inflammatory response, increase the recruitment of pro-inflammatory cells, and secrete cytokines. Oxidative stress results in DNA damage and ultimately promotes tumorigenesis, invasion, and metastasis, affecting tumor response to therapy and other aspects (75, 76).

Chronic pancreatitis is a risk factor for PDAC. Systemic and local chronic inflammation increases the risk of PDAC, and the associated inflammation in the tumor microenvironment contributes to tumor growth and metastasis (77). Chronic inflammation of chronic pancreatitis may also be caused by microbial infection (78). In a mouse model of pancreatic cancer, Kras itself causes spontaneous infiltration of immune cells, and other chronic inflammatory stimuli further accelerate the development of pancreatic cancer (79). In mice with KRAS mutations, pancreatitis that lasts for 4 weeks can cause pancreatic intraepithelial neoplasia (PanIN), which are precancerous lesions (4). In the earliest stages of PDAC formation, an active inflammatory stimulus and fibrotic environment support cancer cell immune response evasion and metastasis to distant organs, which are important factors for cancer cell survival, immune evasion, and metastasis (80).

## Epithelial–Mesenchymal Transition (EMT) Induced by Pancreas-Related Microorganisms and Pancreatic Cancer

Epithelial–mesenchymal transition is a process in which epithelial cells acquire mesenchymal features. In cancer, EMT is related to tumor initiation, invasion, metastasis, and resistance to therapy (81). Chronic inflammation caused by bacterial or viral infections is associated with certain types of cancer, and these microorganisms upregulate the expression of some transcription factors involved in EMT regulation (82). Microbes induce EMT by inducing cell signaling that mediates transcription factor activation via specific transmembrane receptors, and growth factors and microbes share common signaling pathways (82). Microbes promote tumorigenesis by triggering EMT via E-cadherin/β-catenin and inducing epithelial barrier alterations in EMT and tumor-promoting inflammation (83). Opportunistic infections of various pathogens can promote malignant progression. The virulence factor FadA is expressed on the surface of Fusarium sclerotium. It binds and induces phosphorylation/internalization of E-cadherin, thereby disrupting cell-cell junctions. Then the release of β-catenin from the plasma membrane and further activation of the Wnt signaling pathway (phosphorylation/degradation of GSK3β and decomposition of the APC/Axin/GSK3β complex) occur, leading to enhanced EMT and invasion of cancer cells (84). Chronic inflammation associated with long-term microbial infections cause continuous activation of NF-κB and mitogen-activated protein kinase modules as the basis of EMT, which ultimately leads to fibrin production, cancer progression, and metastasis (82). Viruses can induce EMT, and viral infections can lead to activation of intracellular signaling pathways. The main pathogenic viruses are cytomegalovirus, herpes simplex virus, hepatitis B virus, and hepatitis C virus (82). EMT induction in pancreatic ductal epithelial cells represents an early event in PDAC development (85). The EMT process is regulated by complex networks of cytokines, transcription factors, growth factors, signaling pathways, and the tumor microenvironment, exhibiting cancer stem-like properties. The transition of solid cancer cells from the epithelial phenotype to the intercellular phenotype increases their migratory and invasive properties, thus promoting metastasis. EMT and chemoresistance in pancreatic cancer have also been implicated. Inflammation enhances the occurrence and development of pancreatic cancer and promotes EMT in the early stages of PDAC invasion (86). KRAS mutation is a hallmark of PDAC, while EMT is the driving force for its progression (87). Inflammation, EMT, and cancer are tightly linked. The molecular regulatory mechanisms of inflammation and EMT in PDAC during tumor occurrence and progression include the interaction among NF-κB, transforming growth factor β (TGF-β), tumor necrosis factor α (TNF-α) , and signal transducer and activator of transcription 3 (STAT3) signaling pathways. NF-κB is not only a direct and powerful inducer of EMT, but also promotes the mobilization of innate immunity and inflammation, thus building a molecular bridge between inflammation, EMT, and cancer (88). EMT is a key step in PDAC metastasis, allowing polarized motionless epithelial cells to acquire fibroblast-like intercellular abilities such as enhanced motility (89). Each microbial pathogen colonized in pancreatic tissue has the potential to induce EMT and EMT-related pathological changes. For the dynamic process of microbiota-induced EMT in PDAC, elucidating its specific signal transduction pathways and regulatory mechanisms is important for pancreatic cancer occurrence and progression, particularly metastasis.

## Immune Regulation Induced by Pancreas-Related Microorganisms and Pancreatic Cancer

The microbiome plays an important role in the development and maturation of the immune system, destroying tumor immune surveillance processes and promoting pancreatic cancer (90).

A large number of immune cells infiltrate the stroma of PDAC tumor tissues, including T cells, B cells, neutrophils, monocytes/macrophages, and mast cells, which promote the initiation, progression, and immune evasion of PDAC (79, 91). Many different types of immune cells have been found to cause changes in PDAC inflammation (**Table 2**) (16). The activity of these cells and their role in immune regulation during tumor development have not yet been fully elucidated. However, tumor cells seem to alter the activity of immune cells, thus promoting tumor growth and development. The existence of these immune cells in PDAC is related to poor prognosis. Interestingly, some of the most typical immune cells promote tumor development. However, these cells have a tumor-suppressing effect by changing their polarity (92). Infiltration of CD4+ T lymphocytes and CD8+ T lymphocytes are beneficial for improving the prognosis of PDAC patients (93). Infiltration of intratumoral CD4+ Th2 cells in PDAC is associated with reduced survival (94). Foxp3+ Tregs can promote immune escape in PDAC (95).

**Table 2:** Summary of immune cells in the tumor microenvironment of pancreatic adenocarcinoma.

Immune Cell Type	Infiltration and distribution			Related Cytokines	Function	Prognostic factors associated with PDAC	Other known immune functions
	Pancreatitis	Pancreatic ductal intraepithelial neoplasia	PDAC				
Neutrophil	High	Present	Very low	IL-8, C5a, MMP	Promoting inflammation, promoting cancer	No difference in various types of PDAC	Resist pathogens such as bacteria and viruses.
TAM							
M1	High	Present	Present	IFN-γ, IL-12, IL-23, TNF-α	Promoting inflammation, promoting cancer		Participate in th1 type immune response, killing pathogens and tumor cells
M2	Low	Low	High	IL-4, IL-10, IL-13, TGF-β	Immunosuppression	Poor survival rate	Mediates Th2-type immune response and plays a major role
MDSC	Present	Low	High	GM-CSF, VEGF, TGF-β	Immunosuppression, decreased CD8 + T cell infiltration	Poor survival rate	Suppress immune cell response
T cell							
Treg	Present	Low	High	TGF-β, IL-6	Immunosuppression, decreased CD8 + T cell infiltration	Poor survival rate	Immunosuppression and tolerance.
Helper T cell							
Th1	Present	Low	Low	IL-2, IFN-γ	Immune activation	Better survival rate	Assist in the removal of microorganisms in cells
Th2	Present	Low	High	IL-4, IL-5, IL-6, IL-13	Immunosuppression	Poor survival rate	Participate in allergic reactions against
Effector T cell	Present	Low	Low	IFN-γ, TNF-α, IL-2	Antitumor immunity	Better survival rate	Recognize infected cells and bind to cell
Th17 cell				IL-17A,IL-17F,IL22,TNF-α	Promoting inflammation		Activate neutrophils, mediate inflammation, and tumor immunity.
DCs				L12,IL18,cck	Antitumor immunity		Inducing specific CTL anti-tumor immune response.

TAM, tumor-associated macrophage; VEGF, vascular endothelial growth factor; MDSC, myeloid-derived suppressor cell.

The microbiome in the pancreas effectively regulates the tumor immune microenvironment. Bacteria can promote the progression of pancreatic cancer in the mouse PDAC infiltration model, oral antibiotic treatment can reduce the tumor burden by 50%. If the bacteria of the PDAC host are retransplanted into the intestines of mice, the growth of tumors will be accelerated. The microbiome regulates immunogenicity in PDAC and promotes PDAC progression by inducing peritumoral immune suppression. The elimination of microorganisms is related to immunogenic changes in the PDAC tumor microenvironment. Tumor-associated macrophage (TAM) phenotype analysis showed that microbial ablation resulted in a decrease in immune-suppressive CD206+ M2-like TAMs and an increase in M1-like TAMs, which expressed higher MHC II, CD86, TNF-α, IL-12, and IL-6. Anti-microbial treatment resulted in an increased intratumoral CD8:CD4 T cell ratio, promoting Th1 differentiation of CD4+ T cells and CD8+ T cell activation (16). Microorganisms in PDAC produce immune tolerance by activating selective Toll-like receptors (TLRs) in monocytes (96). TLRs, which are the most recognized family of pattern-recognition receptors (PRRs), which are a group of molecular pattern receptors related to pathogens (96). These receptors play a role in the immune response of microbial infections and accelerate tumorigenesis via innate and adaptive immune suppression in PDAC. A variety of PRRs, including TLR3, TLR4, TLR7, TLR9, NLRP3, Dectin-1, and Mincle, are upregulated in PDAC (16, 20). The microbiota also induces the activation of NOD-like receptors (NLRs), which also belong to the PRR family and can recognize different but overlapping microbial components. NOD2 plays a key role in activating NF-κB signaling and forming bacterial communities (97).

Bacterial elimination can also improve the efficacy of immunotherapy targeting checkpoints by upregulating PD-1 expression. To-date, monoclonal antibodies have successfully blocked two checkpoints: cytotoxic T-lymphocyte-associated protein 4 (CTLA-4) and programmed cell death protein 1 (PD-1)/programmed cell death ligand 1 (PD-L1) (98). PD-L1 is expressed in 60 to 90% of tumor cells in human pancreatic cancer. Various studies have shown that multiple pathway-dependent regulation of PD-L1 expression in tumor cells supports immune evasion in pancreatic cancer (99, 100). Studies have found that although PDAC mice have cancer cell-specific CD8+ T cells, the mice do not respond to two immune checkpoint antagonists that promote T cell function (101). However, blocking the PD-L1/PD-1 pathway in vitro can enhance the function of circulating CEA-specific T cells in patients with pancreatic cancer (102). Combination therapy with IL-6 and PD-L1 antibody blockade can reduce tumor progression in a mouse model of pancreatic cancer (103). The combination of antibiotics and PD-1 blockers in PDAC leads to enhanced intratumoral activation of CD4+ and CD8+ T cells, which has synergistic antitumor effects (16). Matson V et al. showed that the commensal microbiome may have a mechanistic effect on antitumor immunity in human cancer patients (104). The gut microbiome uses bile acid as a messenger to control the accumulation of chemokine-dependent liver NKT cells and the mechanism of liver anti-tumor immunity to prevent both primary and metastatic liver tumors (105). Immune checkpoint inhibitors (ICIs) targeting the PD-1/PD-L1 axis induce sustained clinical responses in a sizable minority of cancer patients. We found that primary resistance to ICIs can be attributed to abnormal gut microbiome composition (106). Fecal microbiota transplantation (FMT) from cancer patients who responded to ICIs into germ-free or antibiotic-treated mice ameliorated the antitumor effects of PD-1 blockade, whereas FMT from nonresponding patients failed to do so (106). These data suggest that the endogenous microbiota promotes severe immunosuppressive properties of PDAC, and that the microbiome has the potential to be a therapeutic target in regulating disease progression (16).

In relation to long-term survival (LTS) of PDAC, microorganisms in tumors enhance immune infiltration, showing activated, polyclonal, tumor-specific T cell infiltration (107). Riquelme et al. (17) found that the density of CD3+ and CD8+ T cells in LTS was higher than that in short-term survival (STS), and a higher number of granzyme B + (GzmB) cells were detected in LTS. CD8+ T cells and GzmB+ tissue densities were significantly correlated with microbiome diversity. The diversity of the tumor microbiome contributes to the anti-tumor immune response by promoting the recruitment and activation of CD8+ T cells. The gut microbiome also affects the immune infiltration of pancreatic tumors. Tumors from mice that received fecal microbial transplantation (FMT) from LTS with no evidence of disease (LTS-NED) had higher numbers of CD8+ T cells and higher serum levels of interferon-g (IFN-γ) and interleukin-2 (IL-2), whereas those who received STS FMT had increased CD4+, FOXP3+, and myeloid-derived suppressor cell (MDSC) infiltration. CD8+ T cell depletion blocks the anti-tumor effect induced by LTS-NED FMT, indicating that the beneficial effect of LTS-NED-related gut/tumor bacteria is mediated by CD8 + T cells (17, 108). In short, the gut microbiome can colonize pancreatic tumors, change their overall tumor bacterial composition, and regulate immune function, which ultimately affects the prognosis of PDAC.

## Pancreas-Related Microbial Metabolism and Pancreatic Cancer

Microbial metabolites in the tumor microenvironment affect immune cell differentiation and function. Metabolites produced by some bacteria can promote the production of peripheral regulatory T cells (109). Microbial bile acid metabolites can modulate gut RORγ regulatory T cell homeostasis (110). Microbial-derived short-chain fatty acids can promote the memory potential of antigen-activated CD8+ T cells (111). Bacterial metabolites can also affect the host immune system to suppress inflammation and prevent tumors. Microbial metabolites (e.g. palmitoleic acid, short-chain fatty acids) reduce inflammation by regulating the production of Foxp3 + T cells or reducing IFN-γ produced by T cells (112). The microbiome causes changes in human metabolism, leading to metabolic diseases such as obesity and diabetes, which are important factors in the development of PDAC (30). Geller et al. have experimentally demonstrated that pancreatic colonized bacteria are part of the PDAC tumor microenvironment and may play a key role in mediating chemotherapy resistance. Gemcitabine is a nucleoside analog used to treat pancreatic cancer, lung cancer, breast cancer, or bladder cancer. In pancreatic tumors, γ-Proteus expresses the bacterial enzyme cytidine deaminase (CDDL), which can metabolize gemcitabine (2', 2'-difluorodeoxycytidine) into the inactive form 2', 2'-difluorodeoxyuridine, affecting its therapeutic effect, and the use of antibiotics can eliminate the conversion of this activated form to a non -activated form (21). Three metabolic subtypes were identified (slow proliferating, glycolytic, and lipogenic) from pancreatic cell lines using a metabolomic approach. Then a strong correlation between metabolic and Collisson’s subtype was discovered. The relationship between the influence of the microbiota on metabolism and the different genetic subtypes of PDAC deserves further discussion (113). A study found that in KPC mice and PDAC patients, serum polyamine concentrations were significantly increased. At the early stages of tumorigenesis, there is a strong correlation between microbial changes and release of metabolites that foster host tumorigenesis. These findings may provide a potential, precise, noninvasive tool for early detection of PDAC, which may result in improved the prognosis (114).

## The Diversity of Pancreas-Related Microbiome and Pancreatic Cancer

Recent studies have shown that microorganisms in PDAC are associated with patient survival. Bacterial DNA was extracted from surgically resected PDAC tissues of 68 patients (36 LTS and 32 STS) and classified by 16S rRNA gene sequencing. Microbiome alpha diversity describes the diversity of species or other taxonomic units in the sample, including species richness, which refers to the number of species present in each tumor sample (115). The alpha diversity of the tumor microbiome in LTS is significantly higher than that in STS, and patients with high alpha diversity have significantly extended overall survival, so tumor alpha diversity can be used as a predictor of survival outcomes in patients with surgically resected PDAC. The tumor microbiome is significantly different between LTS and STS. Tumor tissues from LTS patients showed enrichment of Proteobacteria (*Pseudomonas*), *Saccharopolyspora*, and *Streptomyces*, whereas nodominant bacteria were detected in STS patients. In addition, the composition of the intratumoralmicrobiome determines the different metabolic pathways between LTS and STS patients (17, 116).

Gopalakrishnan et al. examined the oral and gut microbiome of melanoma patients undergoing anti-PD-1immunotherapy (n=112). Significant differences were observed in the diversity and composition of the patient gut microbiome of responders (R)versus non-responders (NR). Analysis of patient fecal microbiome samples (n=43, 30R, 13NR) showed significantly higher alpha diversity and relative abundance of *Ruminococcaceae* bacteria in responding patients. Systemic immunity and anti-tumor immunity are enhanced in patients with a favorable gut microbiome, as well as in germ-free mice receiving fecal transplants from responding patients (117). Half et al. analyzed the fecal microbiota of 30 patients with pancreatic adenocarcinoma, 6 patients with pre-cancerous lesions, 13 healthy subjects and 16 with non-alcoholic fatty liver disease, using amplicon sequencing of the bacterial 16S rRNA gene. Fourteen bacterial features discriminated between PC and controls (39). A prospective study collected 85 PC and 57 matched healthy controls (HC) to analyze microbial characteristics by MiSeq sequencing. The results showed that gut microbial diversity was decreased in PC with a unique microbial profile, which partly attributed to its decrease of alpha diversity (118). The tumor microbiota can be influenced by changes in the gut microbiota. The fecal microbes of patients with advanced PDAC were transplanted to mice that had previously been treated with antibiotics. It was observed that tumor growth in the mice that received FMT from LTS-NED donors was significantly slower than that of mice receiving FMT from STS donors or healthy control donors. Tumors from mice with FMT from STS were larger than those from mice with FMT from healthy control donors, suggesting that PDAC-related intestinal/tumor bacteria may play a tumor-promoting effect. The gut/tumor bacteria from patients who had PDAC and survived long-term can inhibit tumor growth, and bacterial elimination can reduce the anti-tumoral efficacy induced by LTS-NED FMT (17). While PC-associated microbial signatures are easily observed, their translation to predictive biomarkers is not straightforward. However, a feasible approach may be to combine several microbial features with other non-invasive biomarkers, such as the serum biomarker CA19-9 which is of limited use in PC detection, or urinary biomarkers currently being investigated, for increased accuracy (39). Various studies have indicated the important role of the gut and tumor microbiome in pancreatic cancer. In the future, microbiome diversity could be used to predict the PDAC survival rate and guide new treatment strategies.

## Conclusion

In summary, the microbiome colonized in pancreatic tissue or tumor is related to the occurrence, development, treatment response, and survival period of pancreatic cancer. The elimination or increase in diversity of the pancreatic microbiome is beneficial for prolonging the survival of PDAC patients, improving the response to chemotherapy drugs, and reducing tumor burden. It is worth noting that the living environment of animals and the sampling errors of human samples will affect the study of microorganisms and should be strictly controlled. Conducting certain clinical trials within an appropriate range helps to understand the relationship between microorganisms and pancreatic cancer, as shown in **Table 3**. The mechanism of action of the pancreatic colonized microbiome in the tumor microenvironment, as well as immunotherapy approaches require further study in order to find more evidence to explain the complex relationship between the pancreatic colonized microbiome and PDAC. Relevant studies targeting the microbiome may provide insight into the mechanisms of PDAC development and progression, improving treatment effectiveness and overall patient prognosis.

**Table 3:** Representative clinicals of microorganisms in pancreatic cancer.

NCT Number	Title	Study Type	Intervention	Recruitment Status	Estimated Enrollment
NCT03809247	Microbial Diversity of Pancreatic Diseases	Observational	Not provided	Not yet recruiting	330
NCT04274972	The Microbiome of Pancreatic Cancer: "PANDEMIC" Study	Observational [Patient Registry]	specimen collection	Recruiting	20
NCT03302637	Oral Microbiome and Pancreatic Cancer	Observational	16S rRNA gene sequencing assay	Completed	735
NCT03891979	Gut Microbiome Modulation to Enable Efficacy of Checkpoint-based Immunotherapy in Pancreatic Adenocarcinoma	Interventional	Drug	Withdrawn	25
NCT03785210	Refractory Primary Hepatocellular Carcinoma or liver Dominant Metastatic Cancer From Colorectal or Pancreatic Cancers	Interventional	Drug	Recruiting	27

## Author Contributions

WZ, KZ, and PZ reviewed literature and originally drafted the manuscript. JZ and CM contributed to edit and embellished the manuscript. XL approved the final version of the manuscript. All authors contributed to the article and approved the submitted version.

## Funding

This work was supported by National Natural Science Foundation of China (No.81802777), Research Project of Shandong Higher Education Research Center (YJKT201953), Shandong province 2018 Professional degree graduate student teaching case library project (SDYAL18049), Shandong province 2018 graduate teacher guidance ability improvement general project (SDYY18073), "Clinical medicine + X" scientific research project of Affiliated Hospital of Qingdao University.

## Conflict of Interest

The authors declare that the research was conducted in the absence of any commercial or financial relationships that could be construed as a potential conflict of interest.

## Abbreviations

CDDL, cytidine deaminase; CTLA-4, cytotoxic T-lymphocyte-associated protein 4; EMT, Epithelial-mesenchymal transition; FMT, fecal microbial transplantation; GFP, green fluorescent protein; GM-CSF, Granulocyte-macrophage Colony Stimulating Factor; GzmB, granzyme B^+^; IFN-γ, interferon-γ; IL-2, interleukin-2; IL-4, interleukin-4; IL-8, interleukin-8; IL-12, interleukin-12; IL-13, interleukin-13; LTS, long-term survival; LTS-NED, LTS with no evidence of disease; MBL, mannose-binding lectin; MDSC, myeloid-derived suppressor cell; MMP, matrix metalloproteinase; MyD88, myeloid differentiation primary response gene 88; NF-κB, nuclear factor-kappa B; NLRs, NOD-like receptors; PanIN, pancreatic intraepithelial neoplasia; PCR, polymerase chain reaction; PDAC, pancreatic ductal adenocarcinoma; PD-1, programmed cell death protein 1; PD-L1, programmed cell death ligand 1; PRRs, pattern-recognition receptors; TAM, Tumor-associated macrophage; TGF-β, transforming growth factor β; TLR, Toll-like receptor; TME, tumor microenvironment; TNF-α, tumor necrosis factor α; STAT3, signal transducer and activator of transcription 3; STS, short-term survival; VEGF, vascular endothelial growth factor; CI, Candida infection; LPS, Lipopolysaccharide; MAPK, mitogen-related protein kinases (MAPK); TAK1, transforming growth factor β activated kinase 1; IKK, IκB (inhibitor of nuclear factor κB) kinase; NKT, natural killer T; R, responders (R); NR, non-responders (NR); PC, Pancreatic Cancer; HC, healthy controls; FadA, Fusobacterium adhesin A.
